# Dr. George Wallington Grabham

**Published:** 1912-08-10

**Authors:** 


Dr. George Wallington Grabham has died at the age of seventy-five at his residence in Witham, Essex. He was formerly Inspector-General of Asylums and Hospitals in New Zealand. Dr. Grabham obtained the M.D.(Lond.) in 1867, having qualified in 1858. He became resident physician and superintendent of the Earlswood Asylum, which appointment he held for fifteen years. Later, Dr. Grabham became physician to the Bradford Infirmary, and also visiting physician to the Bradford Borough Fever Hospital.

